# Engineering of a thermostable esterase Est816 to improve its quorum-quenching activity and the underlying structural basis

**DOI:** 10.1038/srep38137

**Published:** 2016-12-02

**Authors:** Xiwen Liu, Li-chuang Cao, Xin-jiong Fan, Yu-huan Liu, Wei Xie

**Affiliations:** 1 School of Life Sciences, Sun Yat-sen University, 135 W. Xingang Rd., Guangzhou, Guangdong 510275, P. R. China; 2 State Key Laboratory for Biocontrol, Sun Yat-sen University, 135 W. Xingang Rd., Guangzhou, Guangdong 510275, P. R. China; 3 South China Sea Bio-Resource Exploitation and Utilization Collaborative Innovation Center, Sun Yat-sen University, 135 W. Xingang Rd., Guangzhou 510275, P. R. China; 4 School of Basic Medical Sciences, Anhui Medical University, 81 Meishan Rd., Hefei, Anhui 230032, P. R, China

## Abstract

*N*-acyl-homoserine lactones (AHLs) are small diffusible molecules called autoinducers that mediate cell-to-cell communications. Enzymatic degradation of AHLs is a promising bio-control strategy known as quorum-quenching. To improve the quorum-quenching activity of a thermostable esterase Est816, which had been previously cloned, we have engineered the enzyme by random mutagenesis. One of the mutants M2 with double amino acid substitutions (A216V/K238N) showed 3-fold improvement on catalytic efficiency. Based on the crystal structure determined at 2.64 Å, rational design of M2 was conducted, giving rise to the mutant M3 (A216V/K238N/L122A). The *k*_*cat*_/*K*_*M*_ value of the mutant M3 is 21.6-fold higher than that of Est816. Furthermore, activity assays demonstrated that M3 reached 99% conversion of 10-μM *N*-octanoyl-DL-homoserine lactone (C8-HSL) to *N*-octanoyl- DL-homoserine (C8-Hse) in 20 min, in contrast to the 8 h required by wild type Est816. The dramatic activity enhancement may be attributed to the increased hydrophobic interactions with the lactone ring by the mutation A216V, and the reduced steric clashes between the long side chain of L122 and the aliphatic tail of HSL by the mutation L122A, according to the crystal structure. This study sheds lights on the activity-structure relationship of AHL-lactonases, and may provide useful information in engineering AHL-degrading enzymes.

Quorum-sensing (QS) is a cell-to-cell communication process in bacterial community in which they synthesize, release, detect and respond to small signal molecules in a cell density-dependent manner^1^,^2^,^3^. Among the QS signals (also named autoinducers), *N*-acyl-homoserine lactones (AHLs) are one of the most important types, and have been found in more than 70 bacterial species^4^,^5^. AHLs play essential roles in regulating many important biological functions, including bioluminescence, plasmid transfer, motility, antibiotic biosynthesis, biofilm formation, pathogen infection, exotoxin and extracellular degradative enzymes production^6^,^7^. Therefore they are considered as promising targets for the bio-control strategy known as quorum-quenching. With the advantages of generating less resistant phenotypes and being highly specific against bacteria^8^, quorum-quenching has many biotechnological applications in clinic, agriculture and industry, such as reducing antibiotic usage in aquaculture, plant and animal diseases bio-control, waste water treatment and recombinant gene expression in synthetic biology^7^,^9^,^10^,^11^,^12^,^13^.

AHLs are molecules composed of a homoserine lactone ring and are acylated at the amino nitrogen by a saturated or unsaturated fatty acid (usually 4–18 carbons), which may be oxidized at the beta carbon^14^. There are mainly three types of AHL-degrading enzymes, lactonases (EC 3.1.1.−), acylases (EC 3.5.1.−) and oxidoreductases (EC 1). Lactonases and acylases inactivate AHLs in a hydrolysis manner, acting on the lactone bond and the amide linkage, respectively^15^,^16^,^17^,^18^. On the other hand, oxidoreductases oxidize the ω-1, ω-2 and ω-3 carbons of the acyl chain^19^,^20^. Among these enzymes, lactonases are the best characterized and have been a research hotspot in recent years. Expression of AHLs-lactonases in bacteria or plants has shown significant effects on the QS activities, including reducing pathogen virulence^21^,^22^,^23^,^24^, altering biofilm formation^25^, and enhancing plant resistance to pathogen infection^15^.

Quite a few AHLs-lactonases have been identified and characterized. According to the structures, AHL-lactonases can be divided into two superfamilies, the metallo-lactamases and the α/β-hydrolases. The members of the metallo-lactamase superfamily usually have two zinc ions bound to a conserved H*X*H*X*DH motif located at the active site, which play a crucial role in catalysis via a dual Lewis acid mechanism^26^,^27^,^28^. By contrast, a member of the α/β-hydrolase family, AidH from *Ochrobactrum sp*. strain T63, does not contain the H*X*H*X*DH motif and catalyzes the reaction through a metal-independent manner^29^,^30^. In recent years, four Phosphotriesterase-Like Lactonases (PLLs) family members, *Sso*Pox from *Sulfolobus solfataricus*^31^, GKL from *Geobacillus kaustophilus* HTA426^32^, *Sis*Lac from *Sulfolobus islandicus*^33^ and *Vmo*Lac from *Vulcanisaeta moutnovskia*^34^ were shown to inactivate AHLs and were subsequently crystallized. Each structure exhibits the (β/α)_8_-barrel fold and contains two metal cations: zinc and iron for GKL, while iron and cobalt for the others. Meanwhile, some key amino acids affecting the catalytic efficiency or substrate range were identified by structure-based mutagenesis or *in vitro* evolution^32^,^35^,^36^,^37^. These biochemical and crystallographic studies offer great mechanistic insights into the catalytic mechanisms by AHLs-lactonases. However, a better understanding of their detailed structure-function relationships still requires more studies at a molecular level.

In our previous work, a thermostable esterase Est816 was isolated from a Turban Basin metagenomic library, which degrades a wide range of AHLs (C_4_-C_12_)^38^. Besides, it shows good thermostability with a half-life of about 5 months at room temperature. Thermostability is a very attractive property of enzymes for practical applications. Therefore, we chose Est816 as a starting point for further improvement in the present study. The medium-length chained AHL *N*-octanoyl-DL-homoserine lactone (C_8_-HSL), a key signal involved in the QS process of pathogens, was used as the substrate^39^,^40^. Two beneficial mutations were obtained by random mutagenesis and gave rise to the mutant M2, whose crystal structure was solved by X-ray crystallography. Based on the structural analysis, rational design was performed by introducing mutations to the key residues in the substrate-binding pocket of M2. A third beneficial point mutation L122A was obtained, and together with the M2 mutations, the triple mutant was named M3. The degradation efficiencies of low-concentration C_8_-HSL were investigated and compared between the mutant M3 and the wild type (WT), and the structural basis for the activity enhancement was revealed. This study adds to our knowledge on the AHL-degrading enzymes.

## Results and Discussion

### Mutants with improved AHL-degrading activity

A random mutagenesis library containing about 26000 clones (based on Est816) was constructed for mutant screening with improved AHL**-**degrading activity. The diversity of the library was estimated by sequence analyses of twenty randomly picked clones. The error rate of the library was 1.45-nucleoside changes/kb. About 35% of the clones were active and formed transparent zones around the colonies. These clones were tested by the reporter strain *Agrobacterium tumefaciens* KYC55 (pJZ372) (pJZ384) (pJZ410) (*A. tumefaciens* KYC55)^41^, and two clones showed enhanced quorum-quenching activity (or AHL-degrading activity). C8-HSL can be detected by the reporter strain KYC55, and it induces the expression of β-galactosidase, which hydrolyzes X-gal to develop blue color on the plates. When the substrate C8-HSL was hydrolyzed by the enzyme, the agar plates became colorless. Sequence analysis identified two point mutations, A216V and K238N. The combination of these two mutations resulted in the mutant M2.

### Overall Structure

To reveal the structural basis of the activity enhancement, we determined the crystal structure of a truncated version of the mutant M2-SF (M1-V260). For crystallization purposes, the 11 residues at the C-terminus were removed. M2-SF contains 271 residues, with 11 extra amino acids (including the 6× his tag) being appended at the C-terminus. The crystal diffracted to a resolution of 2.64 Å. Each asymmetric unit contains eight monomers, with a Matthew’s coefficient of 2.04 and an estimated solvent content of 39.7%. All chains are almost completely intact except for very few residues at the N- and C-termini of each subunit, and no internal disorders have been observed. The eight monomers form a tetramer of dimers (the AF, BD, CE and GH pairs respectively), in which chains B and D play a central role in mediating the contacts with the other dimers, forming the scaffold of the assembly (Figure S1A). Within the dimer, the Y36-Q44 and E160-V171 helices serve as the dimer interface. The refined model contains a total of 2074 residues and 400 water molecules. The refinement statistics are summarized in Table 1. The two monomers within the dimers resemble each other, and their RMS deviations are normally 0.3–0.5 Å over at least 258 Cα atoms (Figures S1B). The NCS-averaged monomers are nearly identical, but the G-H dimer displays relatively poorer density and higher B-factors (45.3 and 39.6 Å^2^ for each chain respectively), compared to the average of 31.5 Å^2^ for the entire assembly. A noticeable difference is around the E129-N136 fragment (α4, Figure S1B), which forms the “roof” of the cap domain (L122-P187).

**Figure 1:**
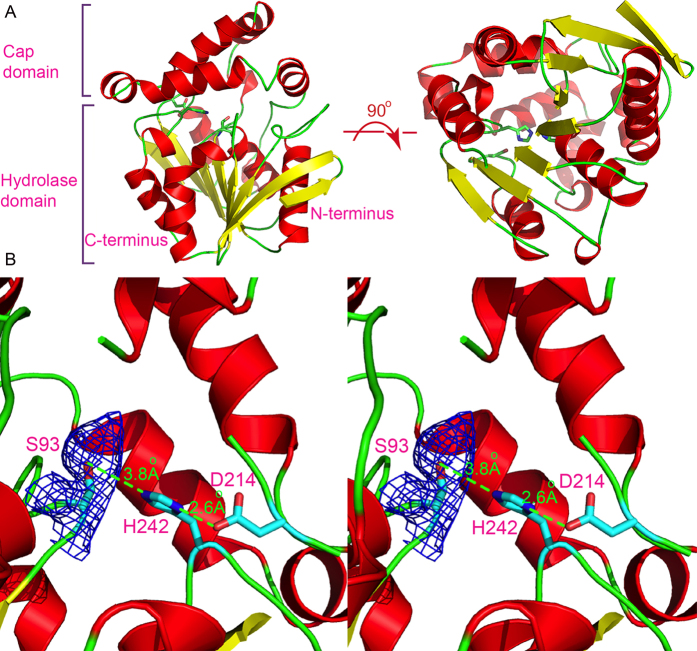
The overall and close-up views of monomer A. (**A**) Two orthogonal views with the helices being colored red and strands colored yellow. The catalytic triad is shown in the ball-and-stick model. (**B**) The detailed interactions of the catalytic triad in cross-eyed stereo views. The possibly phosphorylated form of S93 is shown as suggested by the electron density map. The *F*o-*F*c map density is contoured at 3σ.

The overall structure of each monomer displays the core α/β-hydrolase fold, comprising eight twisted β-strands and eleven α-helices (including three 3_10_-helices), with the sheet being sandwiched by helices on each side (Fig. 1A). β2 (V9-Y16) is the longest strand but runs antiparallel to the other strands, forming backbone interactions with β3 (V20-L25) and β4 (N46-I51). The additional cap domain is inserted between β6 and η3, and packs against the core domain, forming the lid on top of the active site. This cap domain is all helical, consisting of α4-α8. The active site includes the conserved catalytic triad of S93, D214, and H242, and they are located between the core and the cap domain (Fig. 1A). Therefore, Est816 consists of a conserved hydrolase domain and an additional “roof” domain. The preservation of the catalytic triad suggests that a similar hydrolysis mechanism is implemented by Est816, while the variable additional domain may confer substrate diversity.

### Active site

At the active site, the catalytic serine S93 is located on a short loop connecting the tip of α3 (I94-D105) and β5 (A86-G91), which is usually formed by the conserved GXS/D/CXG motif. The OD2 atom of the carboxylate group in D214 forms a hydrogen bond with the ND1 atom of the H242-imidazole ring, and constitutes a part of the proton-shuffling network (Fig. 1B). Of note, no Zn^2+^ is found at the cleavage site. Interestingly, we found that S93 appeared to be in a modified form because extra density in the *F*o-*F*c difference map is connected to the hydroxyl side chain of S93 from each subunit. Additionally, the S93 OG atom is pointing away from H242, and the distance between OG1 and Nε2 is 3.8 Å. These results suggested that S93 is probably in an inhibited form. However, at ~2.6-Å resolution, the identity of the modification group could not be accurately determined. After careful inspection, a phosphoryl group appears to best fit the density. To further investigate the modification nature, we also performed liquid chromatography- electrospray ionization-mass spectrometry (LC-ESI-MS) analysis on the crystallization sample. However, the results showed that S93 was in its native form without any modifications (Figure S2). The origin of the possible modification, or the discrepancy between the crystal structures and the mass spectrometry results is currently unclear. The DNA sequence of the expression construct was double-checked to rule out the possibility of unwanted mutations. Additionally, it is unlikely to be a reaction intermediate, considering the fact that the enzyme displays high activities towards its ester substrates. One possibility was that the functional group of the modification was labile during the pre-treatment process involving chymotrypsin or trifluoric acid (TFA) before the mass-spectrometry analysis. Because we were unable to determine the identity of the electron density, in the final structure we still modeled S93 in its native form.

### Enzymatic characterization

To further improve the activity, rational design was performed based on the crystal structure of M2 (the details of the rational design on M2 are described in the last section). Three separate mutations (P27G/F28N, L122A, V216F) on the basis of M2 were constructed, and tested by *A. tumefaciens* KYC55 in an identical fashion. The triple mutant M2/L122A was named M3 herein.

**Figure 2:**
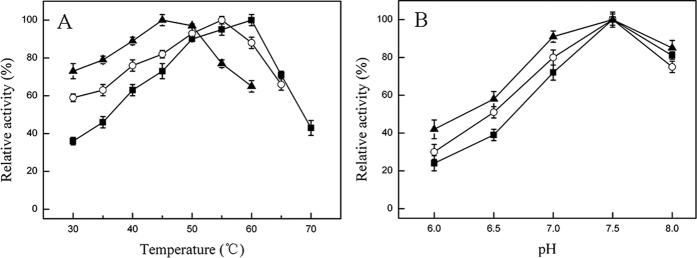
Effects of temperature (**A**) and pH (**B**) on the initial reaction rates of Est816 (■), M2 (○) and M3 (▲). Data points are the average of triplicate measurements, and error bars represent standard deviation.

**Figure 3:**
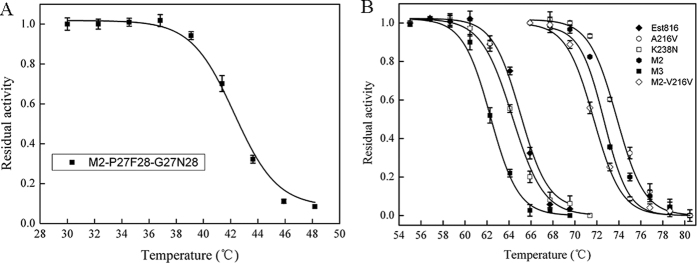
Thermal inactivation curves of Est816 and the mutants. The T_50_ values are 65.2 °C (Est816), 74.1 °C (A216V), 64.3 °C (K238N), 72.2 °C (M2), 42.3 °C (P27G/F28N), 62.2 °C (M3) and 71.2 °C (M2-V216F). Data points are the average of triplicate measurements, and error bars represent standard deviation.

Est816 showed good activities for a wide range of AHLs (C_4_-C_12_)^38^. In this study, C_8_-HSL was chosen as the substrate for activity tests (Figure S3). Est816 acts on C8-HSL in a hydrolysis manner, resulting in the open-ring product *N*-octanoyl-DL-homoserine (C8-Hse) (Figure S3). The optimal temperature (T_opt_) of Est816 is 60 °C (Fig. 2A). The triple mutant M3 decreased the T_opt_ to 45 °C, 15 °C lower than that of the WT (Fig. 2A). Meanwhile, the optimal pHs were not changed significantly by the mutations (Fig. 2B). The effects of the mutations on the thermostability of the protein vary greatly (Table 2, Fig. 3). While the mutation A216V extends the half-life from 20 min (WT) to 18 h, the K238N mutation shows little impact on this property (Table 2). As a result of combined effects, the mutant M2 has a half-life of 12 h at 60 °C. The three mutations introduced by rational design tended to lower the thermostability of M2. The half-life of the mutant M3 is only 10 min (Table 2). Measurements on the T_50_ values of the mutants were consistent with the trend observed in the half-life determination (Fig. 2). The T_50_ value of the mutant A216V is about 9 °C higher than that of WT (74.1 °C *vs* 65.2 °C), while the T_50_ value of the mutant K238N is similar to that of WT (Table 2). The T_50_ value of the mutant M2 is 7 °C higher than that of WT, and the T_50_ value of the mutant M3 is 3 °C lower.

The measured kinetic parameters of Est816 and the mutants are listed in Table 3. The mutation A216V increased the affinity to C_8_-HSL significantly while it did not change the *k*_cat_ value. The mutation K238N improved the *k*_cat_ value but decreased the affinity. Combination of these two mutations resulted in ~3-fold improvement on the *k*_cat_/*K*_M_ value. The mutations P27G/F28N and V216F impaired both the *k*_cat_ and *K*_M_ values. Compared to the mutant M2, introduction of the mutation L122A improved the affinity greatly while decreased the *k*_cat_ value. As a result, the catalytic efficiency of the mutant M3 is 21.6-fold higher than that of Est816. As active as M3 is, its catalytic efficiency is still lower than those of some AHLs-degrading enzymes reported previously^42^. Thus, future studies on this enzyme will mainly be focused on the improvement of its *k*_cat_ value.

### Hydrolysis of low-concentration C_8_-HSL

**Figure 4:**
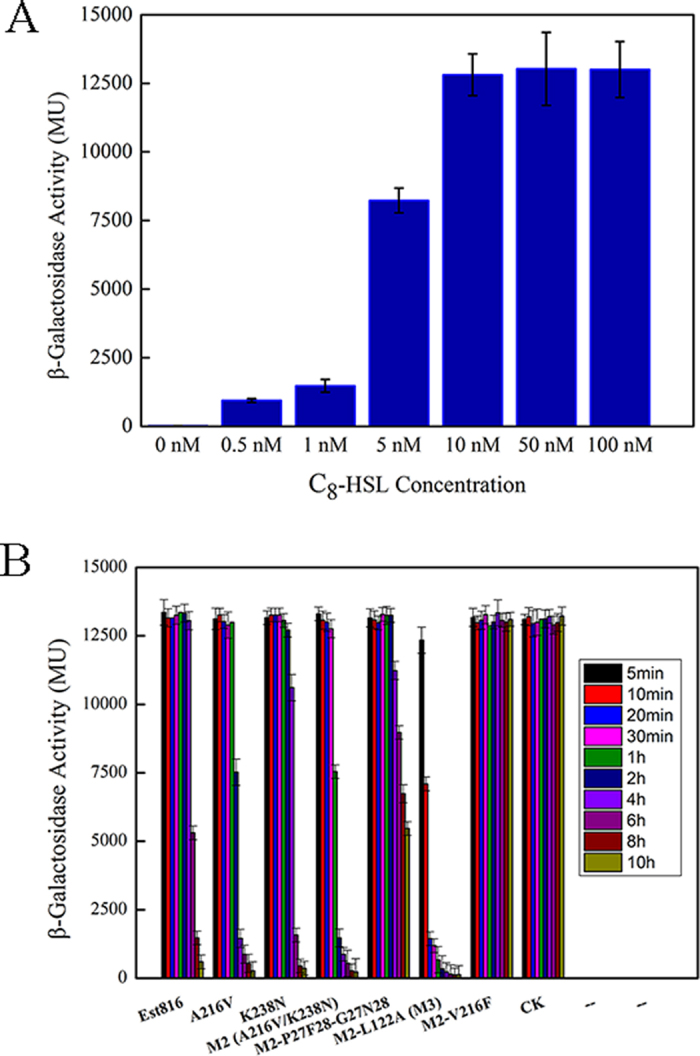
Activity tests using the reported strain. (**A)** C_8_-HSL dose-response curves of *A. tumefaciens* KYC55; (**B**) and time course of C_8_-HSL hydrolysis. The significance was determined by Student’s test (P < 0.05). Data points are the average of triplicate measurements, and error bars represent standard deviation.

AHLs can function as signals at a micromolar or even lower level in the natural environment^42^. Therefore, to assess the application potential of Est816 and the mutants, their degrading abilities towards low-concentration C_8_-HSL (10 μM) were determined. The reactions were performed at 30 °C and pH 7.0, with *A. tumefaciens* KYC55 (pJZ372) (pJZ384) (pJZ410) as the reporter strain. The time required for 99% conversion of the substrate was compared. When no C_8_-HSL was added to the *A. tumefaciens* KYC55 culture, the Miller Units were less than 20 U. Upon the addition of C_8_-HSL at concentrations of 10 nM or higher, the Miller Units reached ~13000 U and remained almost constant. The residual C_8_-HSL concentration in the strain culture was 1 nM when 99% substrate was converted, which induced ~1500 U o-nitrophenyl-β-D-galactopyranoside (oNPG) activity (Fig. 4A). As shown in Fig. 4B, the mutants A216V and K238N hydrolyzed C_8_-HSL more efficiently than the WT, reaching 99% conversion in 4 h and 6 h respectively. In contrast, the WT required 8 h. The combination of the two mutations (M2) shortened the time to 2 h. Remarkably, the extra mutation L122A (M3) further reduced the reaction duration to only 20 min. Therefore the mutant M3 shows greater potential over the WT in inhibiting quorum-sensing. On the other hand, the mutations P27G/F28N and V216F by the rational design dramatically reduced the C_8_-HSL-hydrolyzing activity (Table 3). Although high catalytic efficiency (*k*_cat_/*K*_M_) is always hotly pursued in enzyme engineering, one should be cautious against the pitfalls in considering it as the only standard^43^. From a practical point of view, one should compare the degrading abilities of the enzymes towards AHLs at their working concentrations in the natural environment.

### Comparison to other lactonases

**Figure 5:**
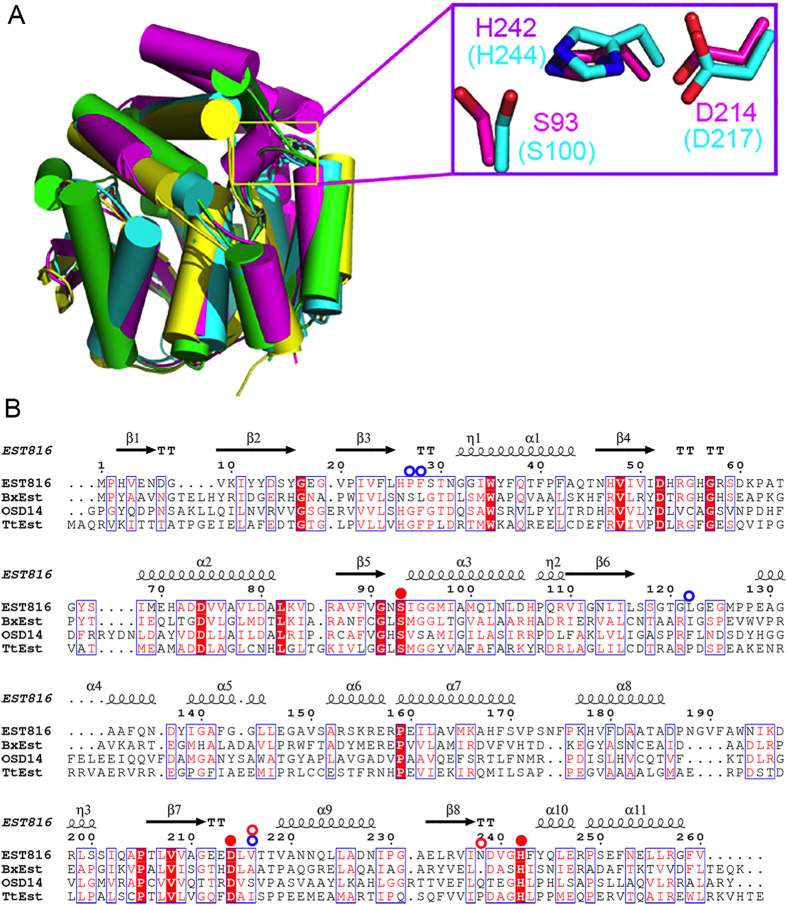
The structure and sequence comparison with orthologs from other organisms. (**A**) Structure overlay of the polypeptide backbones (represented as Cα traces) of Est816-M2 (magenta, PDB 5EGN), BxEst (cyan, PDB 2XUA), TtEst (yellow, PDB 4UHH), as well as OSD14 (green, PDB 3VXK). The catalytic triad in the close-up view is shown in the top-right corner. (**B**) Multiple sequence alignment of Est816 primary sequences as in (**A**). The secondary structure of M2 is drawn on the top. Identical residues in sequences are in magenta and yellow, and similar residues in cyan. The residues of the catalytic triad are shown by the red solid dots, while the positions screened from two rounds of mutagenesis studies were shown by red and blue open dots respectively.

Lactonase is an enzyme widely distributed in eubacteria. A DALI search shows that the closest structural homologues for M2 come from its ortholog from *Burkholderia xenovorans lb400* (BxEst, PDB codes 2XUA)^44^, *Thermogutta terrifontis* (TtEst, PDB code 4UHH)^45^, as well as *Oryza Sativa* (OSD14, PDB code 3VXK)^46^, with decreasing similarities in this order. Est816-M2 shares only 25% sequence identity with its closest structural neighbor BxEst, but the overall structures of these lactonases are conserved (Fig. 5A). The r.m.s. deviation between the core hydrolase domain of Est816-M2 and that of apo-BxEst is 1.2 Å over 184 Cα atoms. Additionally, the critical residues involved in AHL binding are conserved in sequence as indicated by the multiple sequence alignment (Fig. 5B). Notably, the catalytic triads S93-H242-D214 are well conserved, suggesting that these enzymes share a common catalytic mechanism. Structural comparison reveals that the orientation of the catalytic triads is very similar (Fig. 5A).

In contrast, the cap domain shows relative large variations among the aligned structures. Moreover, this domain appears to be more flexible than other regions, as indicated by a much higher B-factor than the rest of the protein. The α4-helix (E129-N137) is most evident, suggesting its structural reorganization upon substrate binding. The r.m.s. deviation of M2 with apo-BxEst is 2.4 Å over 248 Cα atoms. In comparison, the helices of M2 are farther away from the active site than their counterparts, and therefore form larger empty space above the active site, suggesting a less restricted access to the active site. Additionally, the beginning of the cap domain of M2 forms an extra short 3_10_^−^helix, which is not observed in other lactonases.

Another noticeable difference is the dimerization mode of the enzyme. The eight monomers form four dimer pairs (the AF, BD, CE and GH pairs), and bury ~2590 Å^2^ surface area, using the α1 and α7 helices as the interface. The interfacial area is substantially greater than its close homolog such as BxEst, indicating more stable dimers of Est816-M2. Superdex 200 gel-filtration chromatography analysis indicates that M2 forms a species with a molecular weight of ~60 kDa and suggests a dimer in solution (Figure S4), whose theoretical molecular weight is 29.2 kDa for a monomer including the C-terminal 6× his tags. This oligomeric state is in line with the previous reports^28^,^42^.

### Rational design and significance of the mutations

**Figure 6:**
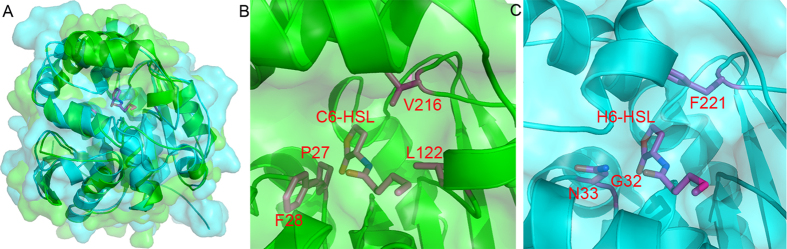
The structure-based rational design to accelerate the reaction rate of Est816. (**A**) Apo-Est816-M2 (green) is superposed onto that of AidH-C6-HSL complex (PDB code 4G8B, cyan). The C_6_-HSL ligand is shown in the ball-and-stick model. (**B**) The close-up view of the substrate-binding pocket of the hypothetical C6-HSL-Est816 complex, with residues tested in the rational design being shown in the ball-and-stick model. (**C**) The close-up view of the substrate-binding pocket of the AidH-C6-HSL complex.

The protein for crystallization was in the apo form, and no ligand was added prior to crystallization. Due to the structural resemblance between Est816-M2 and AidH (an *Ochrobactrum sp*. ortholog, PDB code 4G8B)^30^, we were able to generate a model of M2 complexed with the AHL substrate. By superposition of the apo-M2 structure onto that of the AidH complex, our model shows that the *N*-hexanoyl-DL-homoserine lactone (C_6_-HSL) substrate could fit into the substrate-binding pocket (Fig. 6A). However, the residues forming the binding pocket show quite a few local structural differences. Particularly, C_6_-HSL is recognized by three hydrogen bonds from AidH, which are from the side chains of H248 and N33, as well as the main chain of G32 respectively (Fig. 6C). H248 corresponds to H242 in Est816, a residue of the catalytic triad. However the G32N33 dipeptide is replaced by P27F28 in Est816-M2, which causes relatively large structural perturbation to the local environment (Fig. 6B). On the other hand, close to the lactone moiety, V216 in M2 is substituted by a bulkier residue phenylalanine. V216 is one of the two positions that underwent beneficial mutations obtained from our random screen, and the original residue was an alanine. This suggests that a bulkier residue like valine may be preferred at position 216, possibly to increase the hydrophobic interactions with the lactone ring. Furthermore, the region centering L122 is quite different from that of AidH, and the long side chain of L122 may cause clashes with the aliphatic tail of C_6_-HSL. Therefore, in order to increase the binding affinity of the HSL substrate, we created the L122A, V216F single and the P27G/F28N double mutations on M2 separately, and tested their reaction rates to break down the C_8_-HSL substrate. The results showed that all mutants maintained similar optimal pHs. While the mutation L122A shortened the hydrolysis time of 99% conversion of 10-μM substrate to only 20 min (comparing to 2 h required by M2), the mutations P27G/F28N and V216F dramatically reduced the C_8_-HSL-hydrolyzing activity. These results suggested that position 122 indeed needs to reduce the side chain size, as suggested by 33-fold drop in *K*_M_ and only 7-fold reduction in *k*_cat_ for the L122A mutation (Table 3). On the other hand, greater sizes or flexibility increases at position 216 or 27–28 are unfavorable to the catalytic reaction, with 6.2- and 9-fold reduction in the catalytic efficiencies. Such mutations may bring about large effects on the local structure of the enzyme and are thus more disruptive to its function. We currently do not understand the reason for the 50% activity enhancement caused by the K238N mutation. K238 is on the surface of the protein and it is unlikely for this residue to interact with other residues before or after the mutation, as judged from the structure. However, the performance of an enzyme is a combined result of thermodynamics and kinetics. The mutation may change the surface properties and may be likely responsible for the observed activity changes on *k*_cat_ (Table 3). More investigation is needed to understand the molecular interactions caused by the random mutagenesis. Taken together, the mutagenesis and structural studies provided useful information about the enzymatic properties, and will guide our next-round rational design to further develop Est816 as an efficient quorum-quenching reagent for pathogen outbreak prevention.

## Materials and Methods

### Materials

*Escherichia coli* (*E. coli*) DH5α and pUC19 (TaKaRa, Dalian, China) were used for the construction of random mutagenesis libraries. The *E. coli* strain BL21 (DE3) and the pET-21b (+) plasmid (Novagen, Madison, WI, USA) was used for protein expression. Restriction endonucleases, DNA polymerase and T4 DNA ligase were purchased from Thermo Fisher Scientific (Hudson, NH, USA). *N*-octanoyl-DL-homoserine lactone (C_8_-HSL) and p-nitrophenyl acetate were purchased from Sigma-Aldrich (St. Louis, MO, USA). All other chemicals and reagents were of analytical grade and purchased from commercial sources, unless indicated otherwise.

### Construction and screening of random mutagenesis library

The plasmid pUC19-*est816* (GenBank accession number: JQ996501) was used as the template for the mutagenesis. The screen for more potent hydrolyzing mutants was performed by random mutagenesis, using GeneMorph II Random Mutagenesis Kit (Stratagene, La Jolla, CA, USA) according to the manufacture’s protocol. The error-prone PCR (epPCR) was conducted with the primers 5′-TATAACAGCTATGACCATGATTACGCCGGTACCATG-3′ (forward) and 5′-GAATTCGAGCTCGGTACCCGGAAGCTT-3′ (reverse). The PCR condition was 95 °C for 3 minutes, followed by denaturing at 95 °C for 30 seconds, annealing at 60 °C for 30 seconds, extension at 72 °C for 1 minute for 30 cycles, and finally incubation at 72 °C for 10 minutes. The product was recovered and digested with *Kpn* I and *Bam*H I, and then ligated into pUC19. The ligation product was transformed into *E. coli* DH5α via electroporation. The transformants were cultured at 37 °C overnight on LB-agar plates containing 100 μg/ml ampicillin, 80 μg/ml rhodamine B, 0.1% (v/v) tributyrin, 0.3% (w/v) polyvinyl alcohol and 0.1 mM Isopropyl-β-D-1- thiogalactopyranoside (IPTG). Single colonies that formed transparent zones were picked for further screening according to Schipper *et al*.^47^ with minor modifications. *A. tumefaciens* KYC55 (pJZ372) (pJZ384) (pJZ410) was used as the reporter strain^41^ and the C_8_-HSL concentration was 1.5-fold of the inactivating concentration for WT Est816. In brief, overnight cultures of the clones were mixed with C8-HSL and incubated for 20 h at 37 °C. 5 μl of the supernatant were pipetted on the screening LB-agar. After overnight incubation at 30 °C, development of a blue color indicated quorum sensing, while tests remaining colorless indicated possible enhanced quorum-quenching. The corresponding transformants were considered as positive colonies and their plasmids were subsequently extracted for sequence analysis.

### Site-directed mutagenesis

*In vitro* site-directed mutagenesis was performed by using the TaKaRa MutanBEST Kit (TaKaRa, Dalian, China) following the instructions of the manufacturer. The plasmid pET-21b (+)-*est816* was used as the template. The primers used were listed in SI Table S1. The correctness of the mutants was confirmed by DNA sequencing.

### Cloning, overexpression and purification of the target protein

The full-length *est816/M2* gene and a truncated fragment representing residues M1-V260 (named *est816/M2-sf*) were PCR-amplified using the primers 5′-GCGCGCCATATGCCGCATGTAGAGAACGACGG-3′ (forward), 5′-ATATATGCGGCCGCGGACACCA ATGAAGCTTCTCGA-3′ (reverse for *est816*) and 5′-ATATATGCGGCCGCCACGAAACCCCGCAGAAG-3′ (reverse for *est816/M2-sf*), respectively. Then they were sub-cloned into the expression vector pET-21b (+) using the *NdeI* and *NotI* restriction sites with a C-terminal 6× His tag. The plasmids were transformed into the *E. coli* strain BL21 (DE3) cells for overexpression. The expression and purification of the target proteins were conducted according to the method decreased previously^48^. The purified protein was flash frozen and stored at −80 °C at a concentration of 4 mg/ml for further use. The recombinant proteins for activity analysis were further dialyzed in phosphate buffer (50 mM, pH 7.5), and stored at 4 °C for enzymatic assays.

The molecular masses of the denatured recombinant proteins were determined by using sodium dodecyl sulfate polyacrylamide gel electrophoresis (SDS-PAGE) with protein markers of suitable size. The protein concentrations were quantified by using the CoomassiePlus^TM^ (Bradford) Assay Kit (Thermo Fisher Scientific, Waltham, MA, USA).

### Enzymatic assays and determination of thermostability

The effects of temperature and pH on the initial reaction rates of Est816 and the mutants were determined by using C_8_-HSL as substrate. The substrate was firstly dissolved in methanol and then diluted with phosphate buffer. The reactions were triggered by adding 10 μl diluted enzyme solution into 190 μl substrate solution, and stopped with an equal volume of acetonitrile. The residual C8-HSL and its hydrolysis product were quantified by high-performance liquid chromatography (HPLC) with a C18 reverse-phase column (8.0 × 300 mm, 5 μm, ODS, Japan)^24^. A mixture of acetonitrile/HPLC-grade water containing 1% (w/v) acetic acid (70:30, v/v) was used as the mobile phase at a flow rate of 1.0 ml/min at 30 °C. The absorbance at 210 nm was recorded. The initial reaction rates were determined at time points when no more than 20% of the substrate had been converted^49^. The pH buffers include 50 mM citric acid-sodium citrate buffer (pH 5.0–6.0) and phosphate buffer (pH 6.0–8.0). The optimal temperature was determined in the temperature range of 25 °C–70 °C in a phosphate buffer (50 mM, pH 7.5). The kinetic assay of C_8_-HSL was performed at 30 °C in a lactonase buffer according to the method of Thomas *et al*.^50^.

The thermostability of Est816 and the mutants were analyzed by measuring their half-lives (T_1/2_) at 60 °C and T_50_ values. T_1/2_ is defined as the incubation time inactivating 50% of the initial enzyme activity. T_50_ is defined as the temperature where 50% of the protein is inactivated in 10 min. Samples containing 0.1 mg/ml purified enzymes (50 mM phosphate buffer, pH 7.5) were treated either by incubating for various time intervals at 60 °C, or by heating at different temperatures (typically 30–80 °C) for 10 min. Then the residual activities were quantified by using p-nitrophenyl acetate as substrate based on the method of Fan *et al*.^38^. The T_50_ values were determined by fitting a shifted sigmoid function to the thermal inactivation curves.

### Hydrolysis of low-concentration C_8_-HSL

The wild type and the mutants of Est816 each were tested to break down low concentration of C_8_-HSL (10 μM, 50 mM phosphate buffer, pH 7.0). The reactions were initiated by adding 10 μg/ml purified enzymes into the substrate solution and carried out at 30 °C, 120 rpm. Samples were collected at different time intervals, boiled for 5 min and centrifuged. The concentration of the residual C_8_-HSL in the supernatant was assayed on the base of the method reported previously by using the reporter strain *A. tumefaciens* KYC55^41^. In brief, *A. tumefaciens* KYC55 was cultured in LB overnight at 28 °C and 200 rpm. 100 μl of the culture was mixed with 890 μl of fresh LB medium and 10 μl of sample supernatant. Then the mixture was incubated at 28 °C and 120 rpm for 12 h in 24-well plates. The culture was centrifuged at 14000 *g* for 10 min, resuspended in a 50 mM-phosphate buffer (pH 7.0) and used for β-galactosidase activity analysis^51^. 10 μl of the resuspended mixture was added into 490 μl of oNPG solution (50 mM phosphate buffer, pH 7.0). After being incubated for 10 min at 40 °C, the reaction was stopped by adding 500 μl of 1 M Na_2_CO_3_. The Miller Units were calculated according to the following formula:


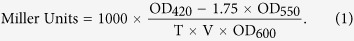


where OD_420_ and OD_550_ were read from the reaction mixture; 1.75 is the coefficient to correct the light scattering at 420 nm caused by cell debris; OD_600_ reflects cell density in the cell suspension; T is the time of the reaction in minutes; V is the volume of culture used in the assay in mLs.

Denatured enzymes were used as the control samples of the hydrolysis assay. Different concentrations of C_8_-HSL (0–1 μM) were used to plot a standard dose-response curve of *A. tumefaciens* KYC55.

### Crystallization and data collection

Crystal hits of M2-SF (M1-V260) were obtained from the Index Screen after two days at 25 °C using the sitting drop vapor diffusion method in a 96-well plate. The reservoir solution contains 28% PEG 3350, 0.1 M sodium acetate (pH 5.6) and 0.2 M sodium chloride. The best crystals were transferred to a cryo-protectant solution containing 20% (v/v) glycerol plus the reservoir solution, and were flash frozen in liquid nitrogen. Native data were collected from frozen crystals at −173 °C using Beamline 17 U (BL17U) at the Shanghai Synchrotron Radiation Facility (SSRF, Shanghai, P. R. China). The space group of the crystal was *P*2_1_ and the crystal diffracted to 2.64 Å with a completeness of 99.8%. Cell content analysis suggested that each asymmetric unit contains eight monomers, with an estimated solvent content of ~40%. All the initial attempts to solve the structure using molecular replacement were failed due to the difficulties in finding enough subunits to build the large assembly. However, a meaningful solution was obtained by molecular replacement using the Balbes webserver^52^ with the coordinates of the *Burkholderia xenovorans lb400* lactonase structure (PDB code 2XUA)^44^ as the search model. This solution contains eight protein molecules in the asymmetric unit. The initial model was further built manually according to the electron-density map using COOT^53^. Multiple cycles of refinement alternating with model rebuilding was carried out by PHENIX. refine^54^ and the NCS refinement was emplyed. The final R-factor was 21.9% (R_free_ = 26.4%) (Table 1). The Ramachandran plot of the final model has 96.50%, 3.35% and 0.15% of the residues in the most favorable, generously allowed and disallowed region. The final model was validated by SFCHECK^55^. The structural figures were produced with PyMOL (www.pymol.org) and the charge distribution on the protein surface was calculated by APBS^56^.

### Tandem mass-spectrometry of Est816

50 μg of the M2-SF mutant was digested by chymotrypsin at a molar ratio of 100 to 1 at 37 °C overnight. The products were detected by SDS-PAGE to confirm that the protein was digested completely. The desalting and concentration of the resulting peptides prior to mass-spectrometry analysis was accomplished by ZipTip pipette tips (Millipore) according to the manufacturer’s instructions. Peptides were analyzed by the LCQ DECA XP ion trap liquid chromatography-mass spectrometer (Thermo). The compound was ionized in the electrospray ionization (ESI) and worked in the MS ^∧^ E mode under default parameters. Data were acquired and analyzed using the Thermo Xcalibur software with a false discovery rate of 4%.

## Additional Information

**Accession Codes**: The atomic coordinates and structure factors have been deposited in the Protein Data Bank with the accession code 5EGN.

**How to cite this article**: Liu, X. *et al*. Engineering of a thermostable esterase Est816 to improve its quorum-quenching activity and the underlying structural basis. *Sci. Rep.* **6**, 38137; doi: 10.1038/srep38137 (2016).

**Publisher's note:** Springer Nature remains neutral with regard to jurisdictional claims in published maps and institutional affiliations.

## Supplementary Material

Supplementary Information

## Figures and Tables

**Table 1:** Data collection and refinement statistics.

Data collection	
Space group	*P*2_1_
Cell dimensions (Å)	
a, b, c (Å)	109.81, 78.54, 116.47
α, β, γ (°)	90.0, 99.4, 90.0
Resolution (Å)	50–2.63 (2.72–2.63)^a^
R_merge_^b^	0.140 (0.518)
I/σ_(I)_	9.4 (2.7)
Completeness (%)	99.8 (99.8)
Redundancy	3.7 (3.7)
Refinement	
Resolution (Å)	44.82–2.64 (2.68–2.64)
No. reflections	57279
R_work_^c^/R_free_^d^	0.219/0.264
No. atoms	
Protein	15784
Water molecules	400
B-factors (Å^2^)	
Protein	31.53
Water molecules	28.41
r.m.s.d.	
Bond lengths (Å)	0.003
Bond angles (°)	0.73

^a^Values in parentheses are for the highest-resolution shell.

^b^R_merge_ = Σ |(I−<I>)|/σ(I), where I is the observed intensity.

^c^R_work_ = Σ_hkl_ ||Fo| − |Fc||/Σ_hkl_ |Fo|, calculated from working data set.

^d^R_free_ is calculated from 5.0% of data randomly chosen and not included in refinement.

**Table 2:** Enzymatic properties of Est816 and the mutants.

Mutants	Optimal temp (°C)	Optimal pH	T_1/2_ at 60 °C	T_50_ (°C)	Time to convert 99% C_8_-HSL^a^
Est816	60	7.5	20 min	65.2	8 h
A216V	60	7.5	18 h	74.0	4 h
K238N	50	8.0	15 min	64.3	6 h
M2 (A216V/K238N)	55	7.5	12 h	72.2	2 h
M2/P27G/F28N	35	8.0	NDb	42.3	24 h
M3 (M2/L122A)	45	7.5	15 min	62.2	20 min
M2/V216F	55	8.0	3 h	71.2	>35 h

^a^Reactions were performed in 50 mM phosphate buffer (pH 7.0) at 30 °C with 10 μM C8-HSL as substrate. ^b^ND = not determined.

**Table 3:** Kinetic parameters of Est816 and the mutants at 30 °C.

Mutants	*K*_M_ (μM)	*k*_cat_ (s^−1^)	*k*_cat_/*K*_M_ (s^−1^ M^−1^)
Est816	1138.2 ± 101.3	0.046 ± 0.004	40.41
A216V	209.9 ± 15.5	0.049 ± 0.003	233.44
K238N	5803.3 ± 289.3	0.400 ± 0.022	68.93
M2 (A216V/K238N)	3492.9 ± 145.7	0.455 ± 0.018	130.26
M2/P27G/F28N	3094.6 ± 198.3	0.064 ± 0.003	20.68
M3 (M2/L122A)	105.2 ± 7.2	0.096 ± 0.007	912.55
M2/V216F	758.7 ± 62.2	0.011 ± 0.001	14.50
