# Supplementary material for: Engineering of a thermostable esterase Est816 to improve its quorum-quenching activity and the underlying structural basis
Source: Sci Rep. 2016 Dec 2;6:38137. doi: 10.1038/srep38137 (PMC5133562; doi:10.1038/srep38137)
Supplement: Supplementary Information [file srep38137-s1.doc]

**Engineering of a thermostable esterase Est816 to improve its quorum-quenching activity and the underlying structural basis**

Xiwen Liu^§, 1, 2^, Li-chuang Cao^§, 1, 3^, Xin-jiong Fan^4^, Yu-huan Liu*^, 1, 3^, Wei Xie*^, 1, 2^

^1^ School of Life Sciences, Sun Yat-sen University, 135 W. Xingang Rd., Guangzhou, Guangdong 510275, P. R. China

^2^ State Key Laboratory for Biocontrol, Sun Yat-sen University, 135 W. Xingang Rd., Guangzhou, Guangdong 510275, P. R. China

^3^ South China Sea Bio-Resource Exploitation and Utilization Collaborative Innovation Center, Sun Yat-sen University, 135 W. Xingang Rd., Guangzhou 510275, P. R. China

^4^ School of Basic Medical Sciences, Anhui Medical University, 81 Meishan Rd., Hefei, Anhui 230032, P. R, China

§These authors contributed equally to this work.

*Correspondence should be addressed to:

Yu-huan Liu: lsslyh@mail.sysu.edu.cn and Wei Xie: xiewei6@mail.sysu.edu.cn.

Tel: 86-20-39332943; Fax: 86-20-39332847

**Supporting information:**

**Supplementary Figure S1-S4, Supplementary Table S1 and Legends.**

**Supplementary materials**

**Figures Legends**

**Supplementary Figure S1.** (A) The overall assembly of Est816. The backbone presentation of the structure superposition of all the eight monomers within the octomer found in the asymmetric unit.Two orthogonal views are shown and the dimer interface is circled by the blue oval on the left panel. （B）Structure overlay of the polypeptide-chain backbones (represented as Cα traces) of Est816-M2 (magenta, PDB 5EGN), BxEst (cyan, PDB 2XUA), TtEst (yellow, PDB 4UHH), as well as OSD14 (green, PDB 3VXK). The region with major differences was circled.

**Supplementary Figure S2.** The LC-ESI mass spectrometry results. (A) The raw data from the fragmentation. The single-charged peaks and molecular weights of the amino-terminal fragments (y) were shown in blue, while those of carboxy-terminal fragments (b) were shown in red. (B) The analysis table of the peptides generated by the MS/MS fragmentation. The left columns listed the fragments carrying one charge (b^+^) and two charges (b^2+^), while the right columns listed the fragments carrying one charge (y^+^) and two charges (y^2+^). The polypeptide sequence containing S93 is VGNSIGGMIAMQL.

**Supplementary Figure S3.** High-Performance Liquid Chromatography (HPLC) elution profiles of C_8_-HSL (A), C_8_-HSL hydrolyzed to C8-Hse (*N*-octanoyl-DL-homoserine) by 10 mM NaOH (B) and C_8_-HSL treated by Est816 (C). The peaks corresponding to C_8_-HSL (retention time of about 4.2 min) and hydrolyzed C_8_-HSL (retention time of about 3.8 min) were indicated by the arrows. (D) The reaction catalyzed by Est816, producing C8-Hse and H^+^.

**Supplementary Figure S4.** Superdex 200 gel-filtration chromatography analysis. The protein standards used were bovine albumin serum (BSA,132 kDa), ovalbumin (43 kDa), carbonic anhydrase (29 kDa). Note that BSA forms a dimer in solution.

**
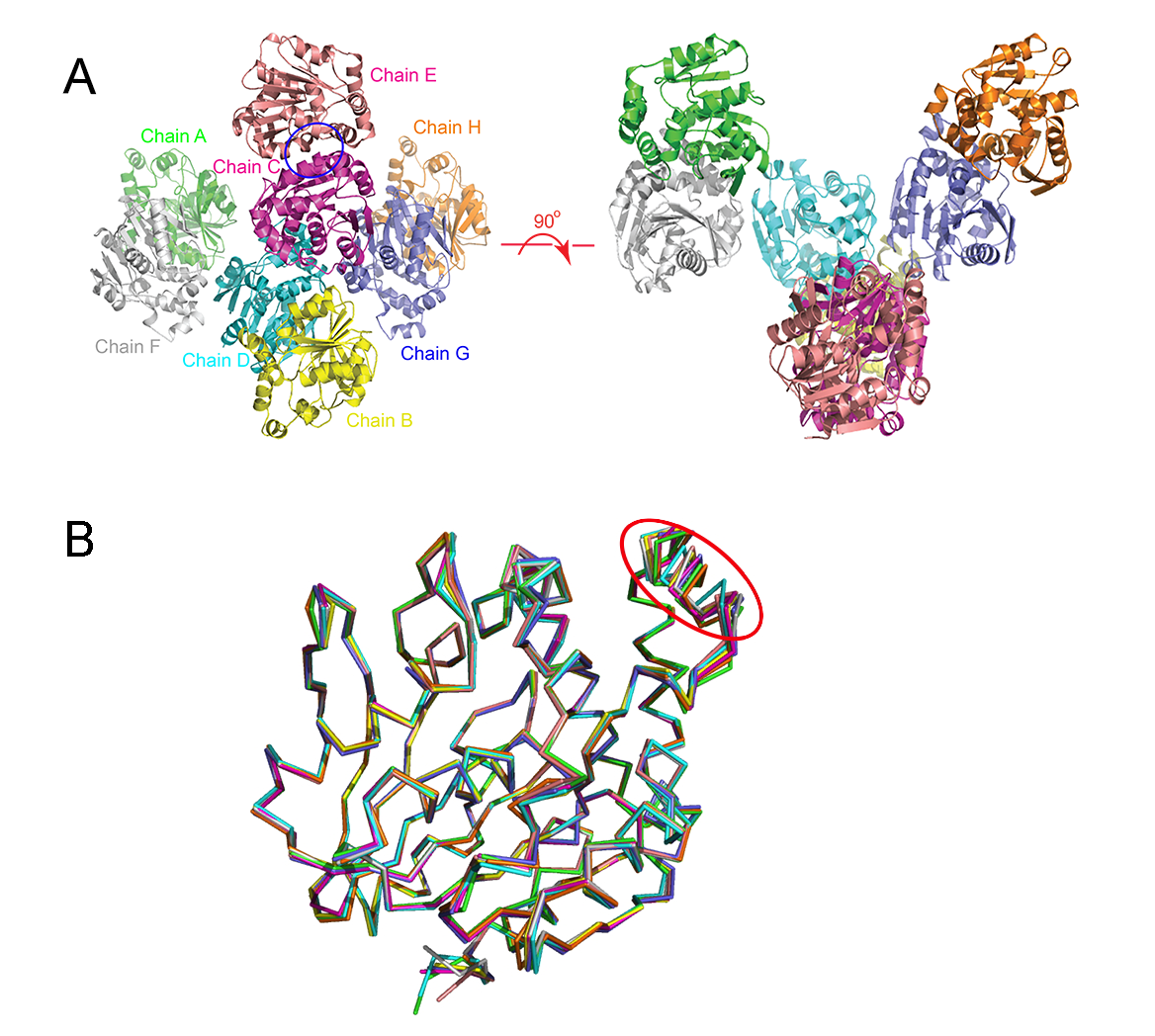
**

**Supplementary Figure S1.**


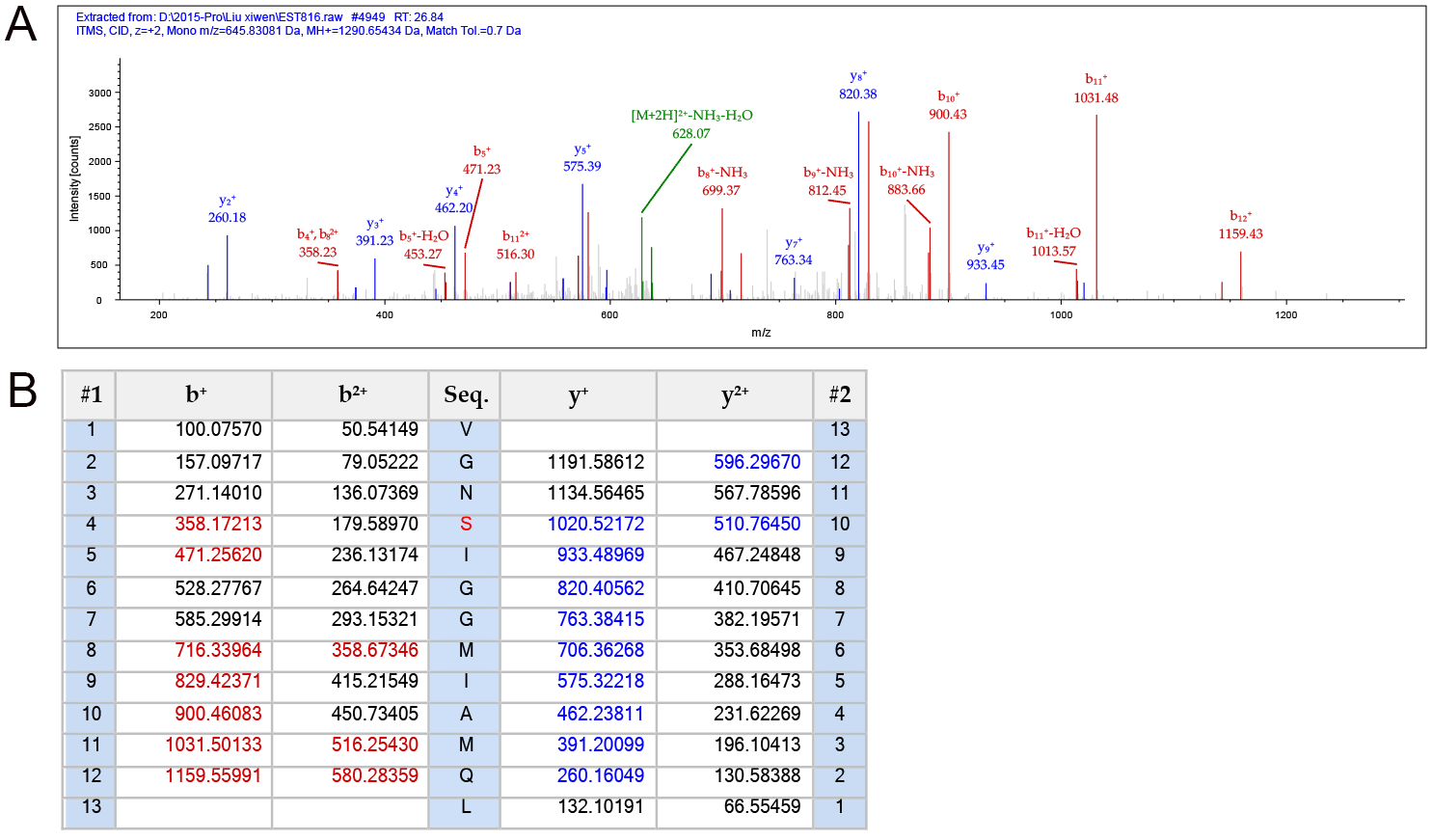


**Supplementary Figure S2.**


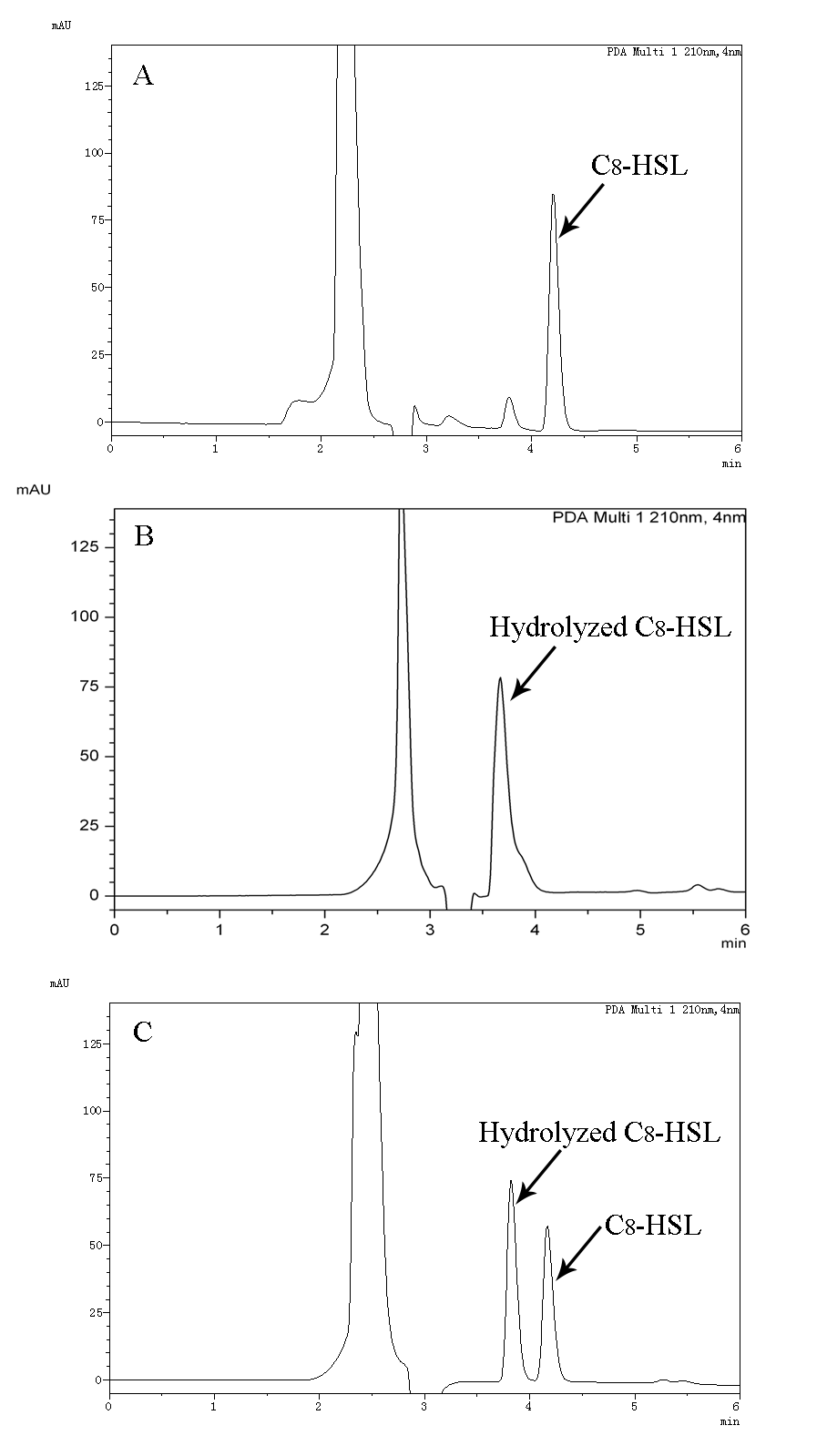


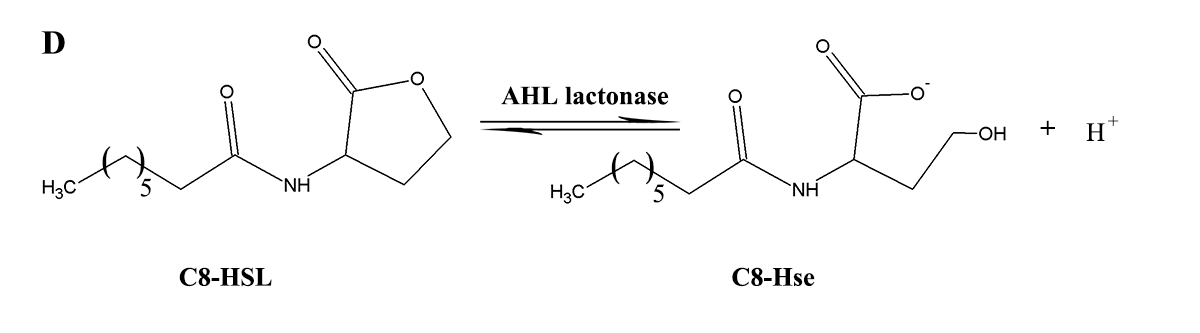


**Supplementary Figure S3.**


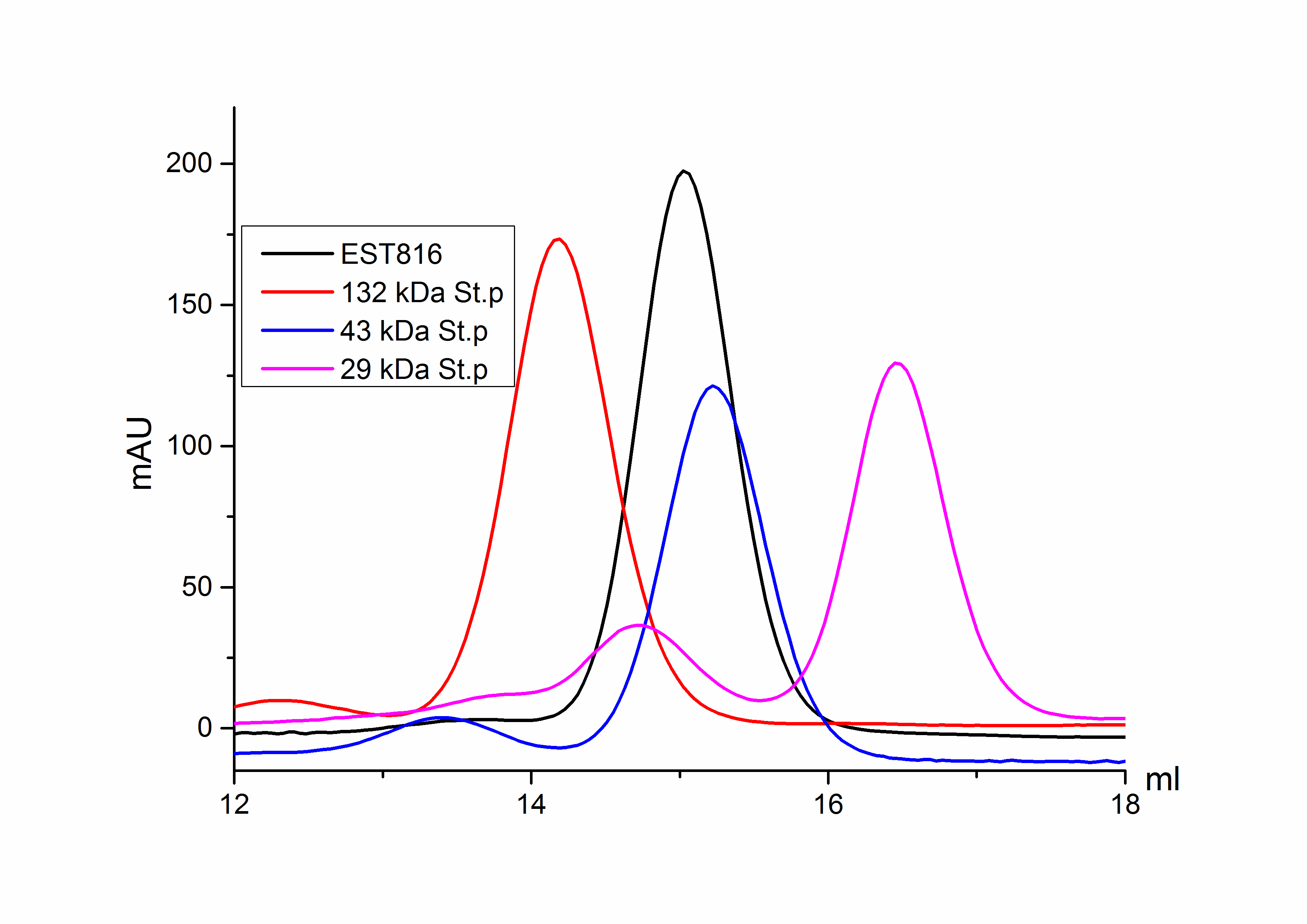


**Supplementary Figure S4.**

**Supplementary Table S1. Primers used to construct the mutants of Est816. The mutated bases were underlined.**

| Mutants | Primers |
| --- | --- |
| P27G/F28N | FP: GGTAACAGCACGAATGGGGGGATCTGGTAC |
|  | RP: ATGAAGGAAGACGATCGGCACGCCC |
| L122A | FP: GCGGGCGAGGGCATGCCGCCCGAAGC |
|  | RP: ACCGGTGCCAGAGCTGAGGATGAGG |
| A216V | FP: GTTGCGCACAAGGCGCATCACGCCA |
|  | RP: GTAGGAGGCGACGTGGGCGGCGC |
| V216F | FP: TTCACGACCGTGGCCAACAATCAAC |
|  | RP: CAGATCTTCTTCTCCGGCGACCACA |
| K238N | FP: AACGATGTCGGGCACTTTTACCAGC |
|  | RP: GATCACGCGCAGCTCCGCACCGGGG |
